# Sphingosylphosphorylcholine blocks ovariectomy‐induced bone loss by suppressing Ca^2+^/calmodulin‐mediated osteoclast differentiation

**DOI:** 10.1111/jcmm.16101

**Published:** 2020-11-23

**Authors:** Ha Young Lee, Kwang Min Cho, Min Kyung Kim, Mingyu Lee, Hun Kim, Cheol Yong Choi, Kyeong Kyu Kim, Joon Seong Park, Hong‐Hee Kim, Yoe‐Sik Bae

**Affiliations:** ^1^ Department of Biological Sciences Sungkyunkwan University Suwon Korea; ^2^ Department of Cell and Developmental Biology BK21 Program and Dental Research Institute Seoul National University Seoul Korea; ^3^ Department of Health Sciences and Technology SAIHST Sungkyunkwan University Seoul Korea; ^4^ Department of Precision Medicine Institute for Antimicrobial Resistance Research and Therapeutics Sungkyunkwan University School of Medicine Suwon Korea; ^5^ Department of Hematology‐Oncology Ajou University School of Medicine Suwon Korea

**Keywords:** calcineurin, osteoclast, osteoporosis, RANKL, sphingosylphosphorylcholine

## Abstract

Osteoporosis is a disease in which bone mineral density decreases due to abnormal activity of osteoclasts, and is commonly found in post‐menopausal women who have decreased levels of female hormones. Sphingosylphosphorylcholine (SPC) is an important biological lipid that can be converted to sphingosine‐1‐phosphate (S1P) by autotaxin. S1P is known to be involved in osteoclast activation by stimulating osteoblasts, but bone regulation by SPC is not well understood. In this study, we found that SPC strongly inhibits RANKL‐induced osteoclast differentiation. SPC‐induced inhibitory effects on osteoclast differentiation were not affected by several antagonists of S1P receptors or pertussis toxin, suggesting cell surface receptor independency. However, SPC inhibited RANKL‐induced calcineurin activation and subsequent NFATc1 activity, leading to decrease of the expression of *Trap* and *Ctsk*. Moreover, we found that bone loss in an experimental osteoporosis mouse model was recovered by SPC injection. SPC also blocked ovariectomy‐induced body weight increase and *Nfatc1* gene expression in mice. We also found that SPC inhibits RANKL‐induced osteoclast differentiation in human macrophages. Since currently available treatments for osteoporosis, such as administration of female hormones or hormone receptor modulators, show serious side effects, SPC has potential as a new agent for osteoporosis treatment.

## 1 INTRODUCTION

Bones are crucial to the framework of vertebrates, and protect the various organs of the body. Bones are also responsible for producing immune cells and storing minerals.^1^ Such bones are maintained by the balance between bone resorption by osteoclasts (OCs) and bone formation by osteoblasts (OBs).^2^ If this balance is broken, a variety of bone diseases occur.^3^ Osteoporosis is a skeletal disorder in which bone resorption occurs abnormally due to the imbalance between bone cells, resulting in weakened and easily fractured bones.^3^ Osteoporosis can be caused by certain diseases, drugs or natural ageing, and, in many cases, is found in post‐menopausal women.^4^, ^5^ Therefore, oestrogen is used to treat osteoporosis,^6^ but causes serious side effects.^7^, ^8^, ^9^

Osteoclasts are involved in bone resorption and are differentiated from bone marrow–derived macrophages (BMDMs) by macrophage colony‐stimulating factor (M‐CSF) and receptor activator of nuclear factor kappa‐Β ligand (RANKL) stimulation.^10^, ^11^ RANKL activates TNF receptor associated factor 6 and mitogen‐activated protein kinase (MAPK) signals through receptor activator of nuclear factor kappa‐Β (RANK) and stimulates transcription factors such as nuclear factor‐κB (NF‐κB), activator protein 1 (AP‐1) and c‐fos to regulate the expression of proteins important for OC differentiation.^12^, ^13^, ^14^ RANKL also regulates nuclear factor of activated T cells, cytoplasmic 1 (NFATc1) activity through calmodulin, which mediates Ca^2+^ signal activation.^13^, ^14^, ^15^ NFATc1 is a master regulator of OC differentiation, which is autoamplified and regulates the expression of several OC‐related proteins such as ATPase H+ transporting V0 subunit D2 (ATP6v0d2), dendritic cell‐specific transmembrane protein (DC‐STAMP), osteoclast stimulatory transmembrane protein (OC‐STAMP), tartrate‐resistant acid phosphatase (TRAP) or cathepsin K (CTSK).^15^, ^16^

Ceramides can be metabolized to sphingosine, which can be converted to sphingosine 1‐phosphate (S1P) via a specific enzyme.^17^ Extracellular S1P stimulates OBs to produce RANKL, which is reported to be involved in promoting OC differentiation.^18^ A previous report demonstrated that sphingosine kinase 1 expression and activity are increased, resulting in increased S1P during RANKL‐induced OC differentiation.^18^ However, intracellular S1P produced by RANKL in OCs has been reported to inhibit OC differentiation.^18^ As such, S1P is known to be involved in the OC and OB balance.^18^ Sphingosylphosphorylcholine (SPC) is a membrane lipid that is converted to S1P by autotoxin.^17^ SPC is a bioactive lipid mediator that acts in a variety of biological processes, including intracellular calcium increase, cell migration, cell growth, proliferation and differentiation.^17^ SPC is known to share receptors with S1P and lysophosphatidic acid (LPA), and to activate intracellular signal transduction through pertussis toxin (PTX)–sensitive G_i_ protein.^19^, ^20^ In addition to membrane receptors, SPC is known to act on the ryanodine receptor or bind to calmodulin to regulate intracellular Ca^2+^ signalling.^21^, ^22^ Ca^2+^‐binding calmodulin is involved in OC formation by regulating cAMP‐response element binding protein (CREB) phosphorylation through Ca^2+^/calmodulin‐dependent protein kinase (CaMK) activity or by regulating NFATc1 nuclear translocation via calcineurin (CaN) activity.^23^ SPC has been reported to bind to calmodulin and regulates calmodulin downstream signals.^22^ A previous report also demonstrated that calmodulin inhibitors block OC formation.^24^ However, it remains to be elucidated whether SPC has an effect on OC generation by RANKL.

In this study, we find that SPC blocks OC differentiation by inhibiting CaN activity and shows beneficial effects in an ovariectomized (OVX)‐osteoporosis mouse model. Based on these results, we propose SPC as a useful agent for the development of new therapeutics to control osteoporosis.

## 2 MATERIALS AND METHODS

### 2.1 Materials

M‐CSF and RANKL were purchased from Peprotech. Alpha‐MEM was purchased from Gibco. FBS was purchased from Access Biologicals. TRAP staining solution kit was purchased from Sigma‐Aldrich. Cell Counting Kit‐8 (CCK‐8) solution was purchased from Dojindo. LPA and S1P receptor antagonists (W146, JTE013, VPC23019, VPC24191 and VPC32183) were purchased from Cayman. PTX was purchased from Calbiochem. All antibodies for phospho‐MAPKs were purchased from Cell Signaling Technology. NFATc1 antibody and total MAPKs antibodies were purchased from Santa Cruz Biotechnology. Lamin B antibody was purchased from Abcam.

### 2.2 OC differentiation and TRAP staining

C57BL/6 mice were purchased from Orient Bio (Seongnam, Korea). All animal experiments were performed in accordance with the guidelines of the Korean Food and Drug Administration. All experiments involving animals received the approval of the Institutional Review Committee for Animal Care and Use at Sungkyunkwan University (Suwon, Korea). Mouse BMDMs were differentiated into OCs using 30 ng/mL M‐CSF and 100 ng/mL RANKL, as previously described.^25^ Peripheral blood was collected from healthy donors. These experiments were approved by Ajou University Hospital's Institutional Review Board for ethics. Human monocytes were isolated from peripheral blood as previously described.^26^ The human monocytes were differentiated into macrophages by adding 30 ng/mL M‐CSF for 3 days. Differentiated human macrophages were further differentiated into OCs by adding 30 ng/mL M‐CSF and 100 ng/mL RANKL for 17 days. The differentiated OCs were stained for TRAP using an acid phosphatase leukocyte diagnostic kit, as described previously.^27^ The TRAP^+^ multinuclear cells (>3 nuclei) with a value > 3 were considered to be OCs.

### 2.3 Cell viability assay

The cell viability assay was performed according to the manufacturer's instructions using the Cell Counting Kit‐8 solution.

### 2.4 RNA isolation and real‐time qPCR

Total RNA from mouse and human OC was isolated using TRIzol reagent (Invitrogen), and cDNA was synthesized using the RT Premix Kit (iNtRON). Mouse femurs and tibiae were rapidly frozen in liquid nitrogen, ground in a mortar and used for RNA isolation using TRIzol reagent. The sequences of primers used include mouse *Nfatc1‐*forward, 5′‐CAACGCCCTGACCACCGATAG‐3′; mouse *Nfatc1‐*reverse, 5′‐GGGAAGTCAGAAGTGGGTGGA‐3′; mouse *Trap* forward, 5′‐CAGTTGGCAGCAGCCAAGGAGGAC‐3′; mouse *Trap*‐reverse, 5′‐TCCGRGCTCGGCGATGGA*CCAGA‐*3′*;* mouse *Atp6v0d2*‐forward, 5′‐TTCTTGAGTTTGAGGCCGAC‐3′; mouse *Atp6v0d2*‐reverse, 5′‐CAGCTTGAGCTAACAACCGC‐3′; mouse *Dc‐stamp*‐forward, 5′‐GGGTGCTGTTTGCCGCTG‐3′; mouse *Dc‐stamp*‐reverse, 5′‐CGACTCCTTGGGTTCCTTGCT‐3′; mouse *Oc‐stamp*‐forward, 5′‐TGGGCCTCCATATGACCTCGAGTAG‐3′; mouse *Oc‐stamp*‐reverse, 5′‐TCAAAGGCTTGTAAATTGGAGGAGT‐3′; mouse *Ctsk*‐forward, 5′‐GGGAGAAAAACCTGAAGC‐3′; mouse *Ctsk*‐reverse, 5′‐ATTCTGGGGACTCAGAGC‐3′; human *NFATc1*‐forward, 5′‐GCATCACAGGGAAGACCGTGTC‐3′; human *NFATc1*‐reverse, 5′‐GAAGTTCAATGTCGGAGTTTCTGAG‐3′; human *CTSK*‐forward, 5′‐TTCTGCTGCTACCTGTGGTG‐3′; human *CTSK*‐reverse, 5′‐GCCTCAAGGTTATGGATGGA‐3′; *GAPDH*‐forward, 5′‐TCCACCACCCTGTTGCTGTA‐3′; and *GAPDH*‐reverse, 5′‐AATGTGTCCGTCGTGGATCT‐3′. Target genes were amplified using a Rotor‐gene Q (2plex on PC) instrument from QIAGEN with SYBR Green qPCR Mix (Biofact).

### 2.5 Nuclear/cytoplasmic fractionation

Mouse BMDMs were differentiated into preOCs (pOCs) using 30 ng/mL M‐CSF and 100 ng/mL RANKL for 2 days. After changing culture medium to serum‐free alpha‐MEM for 4 hours, the cells were stimulated with 30 ng/mL M‐CSF and 100 ng/mL RANKL for 2 hours in the absence or presence of SPC (10 μmol/L). Cells were fractionated using Nuclear and Cytoplasmic Extraction Reagents (Thermo Fisher Scientific) according to the manufacturer's protocol.

### 2.6 Western blot analysis

Cell extracts were prepared in lysis buffer containing 20 mmol/L HEPES (pH 7.2), 10% glycerol, 150 mmol/L NaCl, 1% Triton X‐100, 50 mmol/L NaF, 1 mmol/L Na_3_VO_4_, 10 μg/mL leupeptin, 10 μg/mL aprotinin and 1 mmol/L PMSF. Western blot analysis was conducted as described previously.^27^

### 2.7 Measurement of CaN activity

Cellular CaN phosphatase activity was measured in cell extracts using a CaN cellular activity assay kit (Enzo Life Sciences). In brief, cells were lysed in a lysis buffer containing protease inhibitors and centrifuged. Proteins contained in the supernatant were used for the CaN activity assay using equal amounts (5 μg). Colorimetric measurements were performed at 620 nm.

### 2.8 Isothermal titration calorimetry (ITC) analysis

Isothermal titration calorimetry studies were performed to determine the binding affinities between RANK and RANKL in the presence of SPC using a VP‐ITC microcalorimeter (MicroCal). PBS was used as ITC buffer for both receptor and ligand reservoirs, and all solutions were degassed immediately before reaction. The sample cell was filled with 1.5 mL of 20 μmol/L RANK, and the injection syringe contained 200 μL of 140 μmol/L RANKL. To form a complex with RANK or RANKL, SPC was incubated with each protein in PBS at final concentration of 0.1 mmol/L for 30 minutes at RT. The titration consisted of 20 injections of 10 μL at 180‐second intervals, with the chamber maintained at 25°C. The heat changes of sample cell vs reference cell in chamber were calibrated, and the binding isotherms were fitted to obtain the association constant (*Ka*), enthalpy (Δ*H*) and entropy (Δ*S*). The data were analysed with the Origin 5.0 software (MicroCal).

### 2.9 Ovariectomy (OVX) mouse model and SPC injection

The OVX model is useful to see the effects of ovarian hormone action in menopause‐associated osteoporosis.^28^ For this purpose, control mice, which produce ovarian hormone normally, are needed. Eight‐week‐old C57BL/6 mice (female) were used for OVX, as previously reported.^28^, ^29^ Mice anaesthetized by inhalation anaesthesia had bilateral ovaries removed through the dorsal approach. SPC was injected three times a week via subcutaneous injection (s.c.) from day 1 post‐operation. After 8 weeks, the mice were killed and the femurs separated and used in subsequent experiments.

### 2.10 Microcomputed tomography (μCT) analysis of the distal femur

All mice were killed 8 weeks after surgery, and their bones were fixed in 4% paraformaldehyde solution. Bones were analysed using the SkyScan 1172 μCT scanner (70 kV, 141 μA, 6.92 pixel size; SkyScan). The data were analysed in 1‐mm‐thick areas of distal femurs, starting from 1 mm below the growth plate at thresholds of 75 minimum and 255 maximum. Bone parameters were calculated by a CT Analyzer program (version 1.7; SkyScan), and three‐dimensional images were obtained by CT‐volume software (version 1.11; SkyScan).

### 2.11 Bone histology in OVX mice

All mice were killed 8 weeks after surgery, and their bones were fixed in 4% paraformaldehyde solution for 2 days and embedded in paraffin. For TRAP staining, bones were decalcified in 10% EDTA solution for 5 weeks, with changes of solution 3 times a week before embedding in paraffin. Bones were sectioned into 4 μm slices by a microtome and stained with haematoxylin and eosin for bone histopathological changes or for TRAP to determine osteoclast formation.

### 2.12 Statistical analysis

The results were analysed with GraphPad prism software (GraphPad Software, Inc). Statistical analysis was performed using Student's *t* test. All results are expressed as the mean ± SE. A *P*‐value < .05 was considered statistically significant.

## 3 RESULTS

### 3.1 SPC inhibits RANKL‐induced OC formation

**Figure 1:**
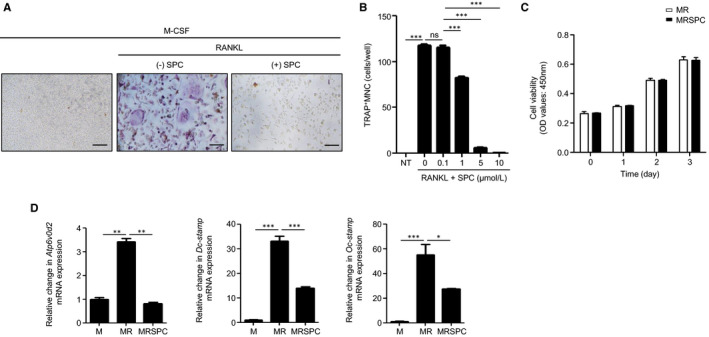
SPC inhibits RANKL‐induced OC formation. A, Mouse BMDMs were stimulated with vehicle (PBS) or SPC (10 μmol/L) in the presence of 30 ng/mL M‐CSF and 100 ng/mL RANKL for 4 d. B, Mouse BMDMs were stimulated with different concentrations of SPC (0, 0.1, 1, 5, 10 μmol/L) in the presence of 30 ng/mL M‐CSF and 100 ng/mL RANKL for 4 d. All cells were subjected to TRAP staining (A, B). TRAP^+^ MNCs (>3 nuclei) were counted (B). C, Mouse BMDMs were stimulated with vehicle (PBS) or SPC (10 μmol/L) in the presence of 30 ng/mL M‐CSF and 100 ng/mL RANKL for 0, 1, 2 and 3 d. After adding the CCK‐8 assay solution, OD values were measured at 450 nm. D, Mouse BMDMs were stimulated with SPC (10 μmol/L) in the presence of 30 ng/mL M‐CSF and 100 ng/mL RANKL for 3 d. The cells were harvested for RNA preparation. qPCR was performed using specific primers for *Atp6v0d2*, *Dc‐stamp*, *Oc‐stamp* and *GAPDH*. Data are representative of at least three independent experiments (A). Data are presented as the mean ± SE of three independent experiments (B‐D). Scale bar, 1 μm. *P < .05, **P < .01, ***P < .001 by Student's *t* test. M, M‐CSF; MR, M‐CSF + RANKL; MRSPC, M‐CSF + RANKL +SPC; MNC, multinuclear cell; ns, non‐significant; NT, not treated

Mouse BMDMs were differentiated into OCs by treatment with 30 ng/mL M‐CSF and 100 ng/mL RANKL for 4 days, and differentiated OCs were identified by TRAP staining (Figure 1A). To examine the effects of SPC on OC formation, we stimulated the BMDMs with SPC (10 μmol/L) in the presence of 30 ng/mL M‐CSF and 100 ng/mL RANKL for 4 days. As shown in Figure 1A, SPC almost completely inhibited RANKL‐induced OC formation. SPC‐induced inhibitory effects on OC formation were concentration‐dependent and showed significant effects at 1‐10 μmol/L (Figure 1B). We then investigated whether the inhibitory effects of SPC on RANKL‐induced osteoclast formation were due to cell death in an in vitro culture system. As shown in Figure 1C, the viability of SPC‐treated cells in the presence of M‐CSF (30 ng/mL) and RANKL (100 ng/mL) was similar to that of vehicle (PBS)‐treated cells in the presence of M‐CSF and RANKL. These results indicate that the inhibitory effects of SPC on RANKL‐induced OC formation are not due to cell death.

The expression of many fusion‐related genes is known to be increased during OC formation.^30^, ^31^ We investigated the effects of SPC on the expression of genes involved in cell fusion during OC formation. The expression of OC fusion‐related genes (*Atp6v0d2*, *Dc‐stamp* and *Oc‐stamp*) was increased by RANKL treatment, and this increase was inhibited upon SPC treatment (Figure 1D). These results suggest that SPC inhibits RANKL‐induced OC formation through the regulation of RANKL‐induced OC fusion‐related gene expression.

### 3.2 SPC‐induced inhibitory effects on OC formation are not mediated by cell surface receptors

**Figure 2:**
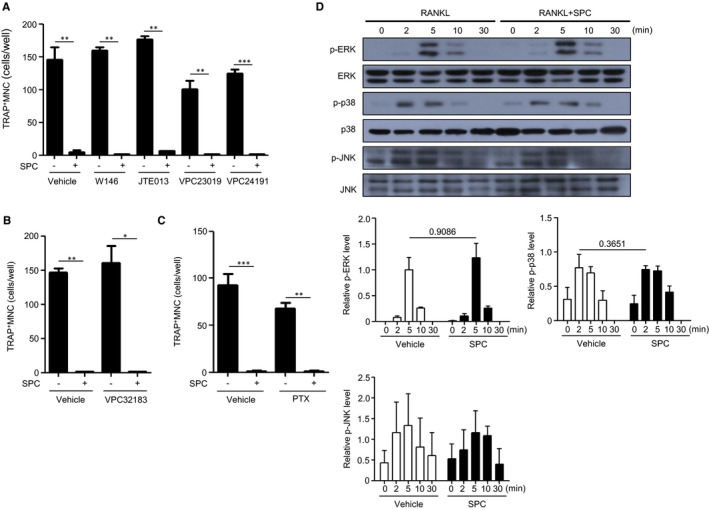
The inhibitory effect of SPC on RANKL‐induced OC formation is independent of cell surface receptors. A and B, Mouse BMDMs were incubated with vehicle (DMSO), different S1P receptor antagonists [W146 (2 μmol/L), JTE013 (5 μmol/L), VPC23019 (10 μmol/L), VPC24191 (10 μmol/L)] (A), or an LPA receptor antagonist [VPC32181 (10 μmol/L)] (B) for 30 min prior to vehicle (PBS) or SPC (10 μmol/L) treatment in the presence of 30 ng/mL M‐CSF and 100 ng/mL RANKL for 4 d. C, Mouse BMDMs were incubated with vehicle (DW) or the G_i_ protein inhibitor PTX (100 ng/mL) for 4 h prior to vehicle (PBS) or SPC (10 μmol/L) treatment in the presence of 30 ng/mL M‐CSF and 100 ng/mL RANKL for 4 d. All cells were subjected to TRAP staining. TRAP^+^ MNCs (>3 nuclei) were counted (A‐C). D, Mouse BMDMs were stimulated with SPC (10 μmol/L) in the presence of 30 ng/mL M‐CSF and 100 ng/mL RANKL for 0, 2, 5, 10 and 30 min. Total cell lysates were separated by SDS‐PAGE. The levels of phosphorylated MAPKs (ERK, p38MAPK and JNK) were determined by Western blot analysis and quantified. Data are presented as the mean ± SE of three independent experiments (A‐C, D *bottom*). Data are representative of three independent experiments (D *top*). *P < .05, **P < .01, ***P < .001 by Student's *t* test

SPC is known to share receptors with S1P or LPA.^32^, ^33^, ^34^ To investigate whether these receptors are involved in the inhibitory effects of SPC on RANKL‐induced OC formation, several antagonists for S1P and LPA receptors were used. Mouse BMDMs were pre‐incubated with these antagonists for 30 minutes according to previous reports,^35^, ^36^, ^37^, ^38^ prior to vehicle or SPC treatment in the presence of 30 ng/mL M‐CSF and 100 ng/mL RANKL. All tested S1P receptor antagonists, such as W146 (S1P_1_ antagonist), JTE‐013 (S1P_2_ antagonist), VPC 23019 and VPC 24191 (S1P_1/3_ antagonists), did not affect the SPC‐induced inhibitory effects on OC formation (Figure 2A). The LPA_1/3_ antagonist VPC32183 also did not affect SPC‐induced inhibitory effects on OC formation (Figure 2B). To investigate the effects of other G_i_ protein–coupled receptors besides the S1P and LPA receptors, we examined the effects of PTX, a G_i_ protein inhibitor. Mouse BMDMs were pre‐incubated with 100 ng/mL of PTX for 4 hours according to a previous report^39^ prior to vehicle or SPC treatment in the presence of 30 ng/mL M‐CSF and 100 ng/mL RANKL. We found that PTX did not affect the inhibitory effects of SPC on OC formation (Figure 2C).

M‐CSF and RANKL are essential factors for the regulation of OC differentiation, and they are known to be involved in cell proliferation, survival, adhesion and cell fusion by affecting signal transduction through MAPK activation.^40^ Thus, we investigated the effects of SPC on M‐CSF‐ and RANKL‐induced MAPK phosphorylation. For this, mouse BMDMs, which are OC precursor cells, were treated with 100 ng/mL RANKL plus 30 ng/mL M‐CSF in the absence or presence of 10 μmol/L SPC. As previously reported,^27^ RANKL plus M‐CSF stimulates MAPK phosphorylation in BMDMs (Figure 2D), showing apparent phosphorylation at 5‐10 minutes (for ERK), 2‐5 minutes (for p38) or 2‐10 minutes (for JNK). However, SPC did not affect RANKL‐induced MAPK phosphorylation. Collectively, the results suggest that the inhibition of OC formation by SPC is not mediated by cell surface receptors or MAPK signalling.

### 3.3 SPC inhibits OC formation via inhibition of CaN activity

**Figure 3:**
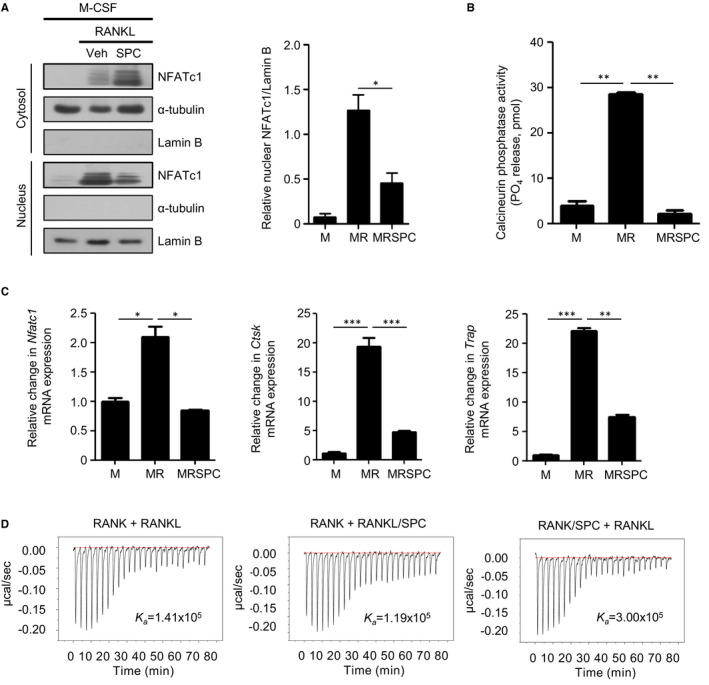
SPC decreases RANKL‐induced CaN activation. A, Mouse pOCs were treated with 30 ng/mL M‐CSF plus 100 ng/mL RANKL in the absence or presence of 10 μmol/L SPC for 2 h. Cytoplasm and nuclear extract were separated by SDS‐PAGE. The levels of NFATc1 were determined by Western blot analysis. B, Mouse BMDMs were treated with 30 ng/mL M‐CSF plus 100 ng/mL RANKL in the absence or presence of 10 μmol/L SPC for 30 min. Cytosolic phosphatase activity of CaN was measured by a CaN cellular activity assay kit. C, Mouse BMDMs were stimulated with SPC (10 μmol/L) in the presence of 30 ng/mL M‐CSF and 100 ng/mL RANKL for 3 d before being harvested for RNA preparation. qPCR was performed using specific primers for *Nfatc1*, *Trap*, *Ctsk* and *GAPDH*. D, The binding affinities between RANK and RANKL in the presence of SPC were determined by using isothermal titration calorimetry. The sample chamber was filled with 1.5 mL of 20 μmol/L RANK, and then, 10 μL of 140 μmol/L RANKL was injected 20 times in 180‐s intervals at 25°C. SPC was pre‐incubated with RANK or RANKL in PBS at a final concentration of 0.1 mmol/L for 30 min at RT. Data are representative of three independent experiments (A *left*, D). Data are presented as the mean ± SE of three independent experiments (A *right*, B, C). *P < .05, **P < .01, ***P < .001 by Student's *t* test. M, M‐CSF; MR, M‐CSF + RANKL, MRSPC, M‐CSF + RANKL + SPC

Since we found that the inhibitory effects of SPC on RANKL‐induced OC formation are independent of cell surface receptors, we investigated whether SPC acts directly on intracellular modulators. According to previous reports, calmodulin is an endogenous intracellular SPC receptor.^22^, ^41^ Calmodulin is known to be involved in RANKL‐induced OC formation through CREB phosphorylation or NFATc1 activation.^23^ Therefore, we investigated the effects of SPC on M‐CSF plus RANKL‐induced CREB phosphorylation and NFATc1 nuclear translocation. Mouse pOCs were treated with M‐CSF (30 ng/mL) plus RANKL (100 ng/mL) in the absence or presence of SPC (10 μmol/L) for 0, 2, 5, 10 and 30 minutes. M‐CSF plus RANKL stimulates CREB phosphorylation, but SPC did not inhibit this phosphorylation (data not shown). Next, we investigated the effects of SPC on M‐CSF plus RANKL‐induced NFATc1 nuclear translocation. pOCs were treated with M‐CSF (30 ng/mL) plus RANKL (100 ng/mL) in the absence or presence of SPC (10 μmol/L) for 2 hours. M‐CSF plus RANKL stimulated NFATc1 nuclear translocation, and SPC inhibited this translocation (Figure 3A *left*). Quantitative analysis showed that SPC significantly inhibits NFATc1 nuclear translocation by M‐CSF plus RANKL at 2 hours (Figure 3A *right*). NFATc1 nuclear translocation is regulated by CaN, which is a calmodulin downstream signalling molecule. Therefore, we investigated the effects of SPC on RANKL‐induced CaN phosphatase activity. Mouse BMDMs were treated with vehicle or SPC (10 μmol/L) in the presence of 100 ng/mL RANKL for 30 minutes. As shown in Figure 3B, RANKL increased CaN activity; however, SPC almost completely abolished RANKL‐induced CaN activity in BMDMs.

NFATc1 is known as a master regulator of osteoclastogenesis and is involved in OC‐specific gene expression.^15^, ^16^, ^42^ We investigated the effects of SPC on the expression of several OC‐related genes (*Nfatc1*, *Trap*, and *Ctsk*) during RANKL‐induced OC formation. RANKL increased the expression of OC‐specific genes; however, SPC strongly inhibited this increased gene expression (Figure 3C). These results suggest that SPC inhibits RANKL‐induced OC formation by inhibiting OC‐specific gene expression through inhibition of NFATc1 nuclear translocation and down‐regulation of CaN activity.

In separate experiments, we also tested whether SPC inhibits RANKL‐induced OC formation through direct binding with RANKL or RANK. Using ITC analysis, although we observed direct binding of RANK and RANKL, we found that SPC does not bind to RANK or RANKL directly (Figure 3D).

### 3.4 SPC administration shows beneficial effects on the OVX‐induced osteoporosis mouse model

**Figure 4:**
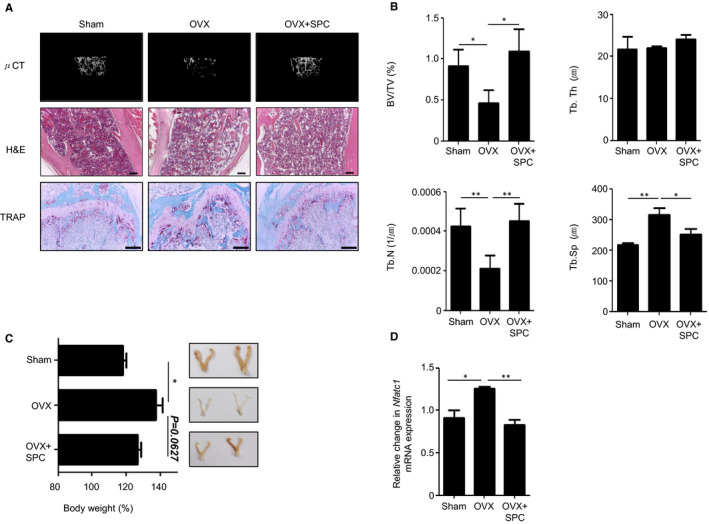
SPC has beneficial effects in ovariectomy‐induced osteoporosis in mice. A‐D, Mice were killed after 8 wk of experiments with s.c. injection of vehicle (PBS) or SPC (2 mg/kg) 3 times per week after OVX surgery. A, Mouse micro‐CT (μCT) analysis, haematoxylin and eosin and TRAP histological staining of mouse femurs of sham, OVX and OVX + SPC mice 8 wk after OVX surgery. B, Structural parameters including bone volume/total volume (BV/TV), trabecular bone number (Tb.N), trabecular separation (Tb.Sp) and trabecular thickness (Tb.Th) were measured. C, Bodyweight of sham, OVX and OVX + SPC mice at 8 wk after OVX surgery (C, *left*). Representative images of uteri in sham, OVX, OVX + SPC mice 8 wk after OVX surgery (C, *right*). D, Femurs and tibias were harvested for RNA preparation. qPCR was performed using specific primers for *Nfatc1* and *GAPDH*. The data are representative of eight mice per group (A, C *right*). Data are mean ± SE of experiments (n = 8) (B, C *left*, D). Scale bar, 100 μm. *P < .05, **P < .01 by Student's *t* test

The OVX mouse model is often used as a post‐menopausal osteoporosis mouse model.^43^ Eight‐week‐old C57BL/6 female mice were divided into three groups: sham, OVX + vehicle and OVX + SPC (n = 8). Mice in the OVX group had their ovaries surgically removed and were injected with vehicle or SPC (2 mg/kg) three times a week for 8 weeks. As shown in Figure 4A, μCT analysis of mouse femurs showed that bone density of OVX mice was decreased compared to sham control mice, and this decrease was dramatically recovered upon SPC injection. Haematoxylin and eosin and TRAP staining analyses showed that OVX surgery elicited increased bone porosity and numbers of OCs in mouse femurs, which changes were markedly attenuated by SPC injection (Figure 4A). OVX mice also showed decreased bone volume compared to trabecular volume (BV/TV), and also decreased trabecular numbers (Tb.N), which were recovered upon SPC injection (Figure 4B). Trabecular separation (Tb.Sp) was also reduced by SPC injection (Figure 4B). Ovariectomized mice have been reported to show increased body weight and decreased uterus size.^44^ We also observed that OVX surgery causes an increase in body weight and decrease in uterus size (Figure 4C). SPC administration blocked these effects (Figure 4C). Femurs and tibiae isolated from sham, OVX and OVX + SPC mice were rapidly frozen with nitrogen, ground in a mortar and used for RNA isolation using TRIzol reagent. We found that the levels of *Nfatc1* mRNA expression were increased in OVX mice, and these levels decreased upon SPC injection (Figure 4D). These results suggest that SPC shows beneficial effects against the OVX‐induced osteoporosis mouse model through regulation of OC differentiation and activation.

### 3.5 SPC inhibits OC formation from human macrophages

**Figure 5:**
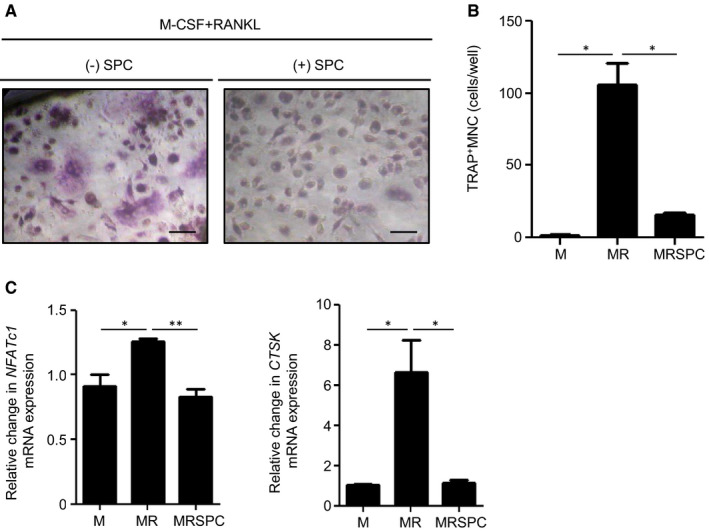
SPC inhibits RANKL‐induced OC formation in human monocyte‐derived macrophages. A and B, Human monocyte‐derived macrophages were stimulated with vehicle (PBS) or 10 μmol/L SPC in the presence of 30 ng/mL M‐CSF and 100 ng/mL RANKL for 17 d. B, Human monocyte‐derived macrophages were stimulated with vehicle or 10 μmol/L SPC in the presence of 30 ng/mL M‐CSF and 100 ng/mL RANKL for 17 d. All cells were subjected to TRAP staining. TRAP^+^ MNCs (>3 nuclei) were counted (A, B). C, Human monocyte‐derived macrophages were stimulated with 10 μmol/L SPC in the presence of 30 ng/mL M‐CSF and 100 ng/mL RANKL for 17 d before being harvested for RNA preparation. qPCR was performed using specific primers for *NFATc1*, *CTSK* and *GAPDH*. Data shown are representative of at least three independent experiments (A). Data are presented as the mean ± SE of three independent experiments (B, C). Scale bar, 1 μm. *P < .05, **P < .01 by Student's *t* test. M, M‐CSF; MR, M‐CSF + RANKL, MRSPC, M‐CSF + RANKL + SPC

To investigate the effects of SPC on human OC formation, we used human monocyte‐derived macrophages. Human macrophages were obtained by treatment of primary human monocytes with 30 ng/mL M‐CSF for 3 days. Differentiated human macrophages were incubated with 30 ng/mL M‐CSF and 100 ng/mL RANKL for 17 days to differentiate into OCs, which were identified by TRAP staining. When 10 μmol/L SPC was administered under OC‐forming conditions, RANKL‐induced OC formation was inhibited by SPC treatment (Figure 5A,B). RANKL‐induced expression of OC‐specific genes (*NFATc1*and *CTSK*) was also significantly inhibited by SPC treatment (Figure 5C). These results indicate that SPC can inhibit OC formation in humans as well as in mice, suggesting a potential beneficial role of SPC on human osteoporosis.

## 4 DISCUSSION

SPC is an important bioactive lipid that mediates a variety of biological processes.^17^ SPC can be converted to S1P, another important bioactive lipid, by autotoxin.^17^ Previous studies have reported that S1P is involved in the regulation of bone formation and resorption in bone metabolism in addition to various cellular responses.^18^ Although SPC has been reported to modulate the activity of pre‐OB cell lines,^45^ little has been reported about its effects on OCs. In this study, we investigated the effects of SPC on OC formation and the therapeutic effects of SPC in an osteoporosis mouse model. SPC inhibits RANKL‐induced OC formation in a concentration‐dependent manner (Figure 1). Although SPC is known to share receptors with S1P or LPA, antagonists for the S1P and LPA receptors showed no effects on the inhibitory effects of SPC on RANKL‐induced OC formation (Figure 2A,C). SPC is also known to act through the PTX‐sensitive G_i_ protein, but PTX did not affect the SPC‐induced inhibition of RANKL‐induced OC formation (Figure 2C). We also found SPC does not bind to RANK or RANKL directly (Figure 3D).Taking our results together, SPC‐induced inhibitory effects on OC generation do not appear to be mediated by cell surface receptors, including the S1P receptors, LPA receptors and RANK/RANKL. A previous report on calmodulin (CaM) as a known intracellular SPC receptor^22^ led us to test the effects of SPC on CaM‐mediated signalling. CaM is known to activate CaMK or CaN by binding to Ca^2+^.^23^ SPC inhibits Ca^2+^ binding to CaM by binding to CaM and inhibits CaMK or CaN activity downstream of CaM.^22^ SPC did indeed inhibit CaN activation by RANKL and inhibited *Nfatc1* gene expression (Figure 3A,B). However, SPC did not inhibit CaMK activity induced by RANKL (data not shown). Rather, CREB phosphorylation was observed to further increase upon SPC treatment (data not shown). Thus, it appears that SPC strongly inhibits phosphatase activity but not kinase activity in CaM downstream signalling induced by RANKL, although the molecular details are yet unknown. Collectively, our results suggest that SPC shows inhibitory effects on OC differentiation by modulating the CaM‐mediated pathway and not by binding to cell surface receptors.

Osteoporosis is a bone disease commonly found in post‐menopausal woman. Oestrogen is a representative female hormone mainly produced in ovaries and decreases with age or in post‐menopausal woman. Oestrogen is known to induce the apoptosis of OCs involved in bone resorption, and thus, it has been reported that osteoporosis is alleviated by oestrogen treatment in an osteoporosis animal model.^46^, ^47^ For this reason, in many cases, ovarian resection is used as a model for post‐menopause osteoporosis. Oestrogen is known to inhibit the accumulation of ceramides, and ceramide levels have been reported to be increased in older and post‐menopausal woman.^48^ Ceramides can be metabolized to S1P by ceramidase and S1P phosphatase, and S1P stimulates OBs to promote RANKL production and to promote OC differentiation.^17^, ^18^ OCs are known to produce autotoxin,^49^ which can convert SPC to S1P. Based on previous literature, it is reasonable to assume that as autotoxin increases, the levels of SPC decrease, and eventually, osteoporosis will worsen as SPC decreases. In support of this hypothesis, in this study, we demonstrated that SPC shows beneficial effects against OVX‐induced osteoporosis. SPC also reverses the effects of OVX on body weight and uterine size (Figure 4C). To the best of our knowledge, there has been no report on the oestrogenic effects of SPC. Therefore, it is very difficult to explain how SPC reverses OVX‐induced changes in body weight and uterine size. Detailed mechanisms of action of SPC on the OVX model should be examined in the future.

In conclusion, the results of this study show that SPC is effective in the treatment of mouse osteoporosis by inhibition of OC differentiation. Since SPC also showed inhibitory effects on human OC differentiation, we suggest that SPC can be used as a new therapeutic agent for the treatment of osteoporosis, which can avoid the side effects of currently used treatments such as oestrogen.

## CONFLICT OF INTEREST

The authors confirm that there are no conflicts of interest.

## AUTHOR CONTRIBUTION

**Ha Young Lee:** Conceptualization (equal); Data curation (equal); Formal analysis (equal); Investigation (equal); Writing‐original draft (equal); Writing‐review & editing (equal). **Kwang Min Cho:** Conceptualization (equal); Data curation (equal); Formal analysis (equal); Investigation (equal); Writing‐original draft (equal). **Min Kyung Kim:** Data curation (supporting); Investigation (supporting). **Mingyu Lee:** Conceptualization (supporting); Data curation (supporting); Investigation (supporting). **Hun Kim:** Data curation (supporting); Formal analysis (supporting); Investigation (supporting). **Cheol Yong Choi:** Conceptualization (supporting); Writing‐review & editing (supporting). **Kyeong Kyu Kim:** Formal analysis (supporting); Investigation (supporting). **Joon Seong Park:** Conceptualization (supporting); Resources (supporting). **Hong‐Hee Kim:** Conceptualization (supporting); Data curation (supporting); Resources (supporting). **Yoe‐Sik Bae:** Conceptualization (equal); Funding acquisition (lead); Project administration (lead); Supervision (lead); Writing‐original draft (supporting); Writing‐review & editing (equal).

## Data Availability

The data that support the findings of this study are available from the corresponding author upon reasonable request.

## References

[1] Raisz LG , Kream BE , Lorenzo JA , et al. Metabolic bone disease In: Davies TF, Larsen PR, Kronenberg HM, eds. Williams Textbook of Endocrinology. Philadelphia, PA: W.B. Saunders; 2002:1373.

[2] Matsuo K , Irie N . Osteoclast‐osteoblast communication. Arch Biochem Biophys. 2008;473:201‐209. 18406338 10.1016/j.abb.2008.03.027

[3] Zaidi M . Skeletal remodeling in health and disease. Nat Med. 2007;13(7):791‐801. 17618270 10.1038/nm1593

[4] Raisz LG . Pathogenesis of osteoporosis: concepts, conflicts, and prospects. J Clin Invest. 2005;115:3318‐3325. 16322775 10.1172/JCI27071 PMC1297264

[5] Khosla S , Melton LJ 3rd , Riggs BL . The unitary model for estrogen deficiency and the pathogenesis of osteoporosis: is a revision needed? J Bone Miner Res. 2011;26:441‐451. 20928874 10.1002/jbmr.262 PMC3179298

[6] Stepan JJ , Hruskova H , Kverka M . Update on menopausal hormone therapy for fracture prevention. Curr Osteoporos Rep. 2019;17:465‐473. 31741221 10.1007/s11914-019-00549-3 PMC6944675

[7] Manson JE , Hsia J , Johnson KC , et al. Estrogen plus progestin and the risk of coronary heart disease. N Engl J Med. 2003;349:523‐534. 12904517 10.1056/NEJMoa030808

[8] Colditz GA , Hankinson SE , Hunter DJ , et al. The use of estrogens and progestins and the risk of breast cancer in postmenopausal women. N Engl J Med. 1995;332:1589‐1593. 7753136 10.1056/NEJM199506153322401

[9] Drugs.com . 2018. Estradiol side effects. http://www.drugs.com/sfx/extradiol‐sieeffects.html. Accessed December 3, 2018.

[10] Suda T , Takahashi N , Udagawa N , Jimi E , Gillespie MT , Martin TJ . Modulation of osteoclast differentiation and function by the new members of the tumor necrosis factor receptor and ligand families. Endocr Rev. 1999;20:345‐357. 10368775 10.1210/edrv.20.3.0367

[11] Teitelbaum SL . Bone resorption by osteoclasts. Science. 2000;289:1504‐1508. 10968780 10.1126/science.289.5484.1504

[12] Lee K , Seo I , Choi MH , Jeong D . Roles of mitogen‐activated protein kinases in osteoclast biology. Int J Mol Sci. 2018;19(10):3004. 10.3390/ijms19103004 PMC6213329 30275408

[13] Yavropoulou MP , Yovos JG . Osteoclastogenesis–current knowledge and future perspectives. J Musculoskelet Neuronal Interact. 2008;8:204‐216. 18799853

[14] Walsh MC , Choi Y . Biology of the RANKL‐RANK‐OPG system in immunity, bone, and beyond. Front Immunol. 2014;5:511. 25368616 10.3389/fimmu.2014.00511 PMC4202272

[15] Lieben L , Carmeliet G . The involvement of TRP channels in bone homeostasis. Front Endocrinol. 2012;3:99. 10.3389/fendo.2012.00099 PMC3422722 22934090

[16] Kuroda Y , Matsuo K . Molecular mechanisms of triggering, amplifying and targeting RANK signaling in osteoclasts. World J Orthop. 2012;3:167‐174. 23330071 10.5312/wjo.v3.i11.167 PMC3547110

[17] Nixon GF , Mathieson FA , Hunter I . The multi‐functional role of sphingosylphosphorylcholine. Prog Lipid Res. 2008;47:62‐75. 18042469 10.1016/j.plipres.2007.11.001

[18] Ryu J , Kim HJ , Chang EJ , Huang H , Banno Y , Kim HH . Sphingosine 1‐phosphate as a regulator of osteoclast differentiation and osteoclast‐osteoblast coupling. EMBO J. 2006;25:5840‐5851. 17124500 10.1038/sj.emboj.7601430 PMC1698879

[19] Okajima F , Kondo Y . Pertussis toxin inhibits phospholipase C activation and Ca^2+^ mobilization by sphingosylphosphorylcholine and galactosylsphingosine in HL60 leukemia cells. Implications of GTP‐binding protein‐coupled receptors for lysosphingolipids. J Biol Chem. 1995;270:26332‐26340. 7592844 10.1074/jbc.270.44.26332

[20] Lee HY , Lee SY , Kim SD , et al. Sphingosylphosphorylcholine stimulates CCL2 production from human umbilical vein endothelial cells. J Immunol. 2011;186:4347‐4353. 21368227 10.4049/jimmunol.1002068

[21] Betto R , Teresi A , Turcato F , et al. Sphingosylphosphocholine modulates the ryanodine receptor/calcium‐release channel of cardiac sarcoplasmic reticulum membranes. Biochem J. 1997;322:327‐333. 9078280 10.1042/bj3220327 PMC1218195

[22] Kovacs E , Liliom K . Sphingosylphosphorylcholine as a novel calmodulin inhibitor. Biochem J. 2008;410:427‐437. 17979830 10.1042/BJ20071019

[23] Sato K , Suematsu A , Nakashima T , et al. Regulation of osteoclast differentiation and function by the CaMK‐CREB pathway. Nat Med. 2006;12:1410‐1416. 17128269 10.1038/nm1515

[24] Ang ESM , Zhang P , Steer JH , et al. Calcium/calmodulin‐dependent kinase activity is required for efficient induction of osteoclast differentiation and bone resorption by receptor activator of nuclear factor kappa B ligand (RANKL). J Cell Physiol. 2007;212:787‐795. 17477372 10.1002/jcp.21076

[25] Lee Y , Kim HJ , Park CK , et al. MicroRNA‐124 regulates osteoclast differentiation. Bone. 2013;56:383‐389. 23867221 10.1016/j.bone.2013.07.007

[26] Gupta N , Barhanpurkar AP , Tomar GB , et al. IL‐3 inhibits human osteoclastogenesis and bone resorption through downregulation of c‐Fms and diverts the cells to dendritic cell lineage. J Immunol. 2010;185:2261‐2272. 20644169 10.4049/jimmunol.1000015

[27] Oh E , Lee HY , Kim HJ , et al. Serum amyloid A inhibits RANKL‐induced osteoclast formation. Exp Mol Med. 2015;47:e194. 26563612 10.1038/emm.2015.83 PMC4673470

[28] Souza VR , Mendes E , Casaro M , Antiorio ATFB , Oliveira FA , Ferreira CM . Description of ovariectomy protocol in mice. Methods Mol Biol. 2019;1916:303‐309. 30535707 10.1007/978-1-4939-8994-2_29

[29] Anginot A , Dacquin R , Mazzorana M , Jurdic P . Lymphocytes and the Dap12 adaptor are key regulators of osteoclast activation associated with gonadal failure. PLoS One. 2007;2:e585. 17611620 10.1371/journal.pone.0000585 PMC1899087

[30] Ishii M , Saeki Y . Osteoclast cell fusion: mechanisms and molecules. Mod Rheumatol. 2008;18:220‐227. 18425565 10.1007/s10165-008-0051-2

[31] Lee S‐H , Rho J , Jeong D , et al. v‐ATPase V0 subunit d2‐deficient mice exhibit impaired osteoclast fusion and increased bone formation. Nat Med. 2006;12:1403‐1409. 17128270 10.1038/nm1514

[32] Meyer zu Heringdorf D , Jakobs KH . Lysophospholipid receptors: signalling, pharmacology and regulation by lysophospholipid metabolism. Biochim Biophys Acta. 2007;1768:923‐940. 17078925 10.1016/j.bbamem.2006.09.026

[33] Takuwa Y , Takuwa N , Sugimoto N . The Edg family G protein‐coupled receptors for lysophospholipids: their signaling properties and biological activities. J Biochem. 2002;131:767‐771. 12038970 10.1093/oxfordjournals.jbchem.a003163

[34] Ge D , Yue HW , Liu HH , Zhao J . Emerging roles of sphingosylphosphorylcholine in modulating cardiovascular functions and diseases. Acta Pharmacol Sin. 2018;39:1830‐1836. 30050085 10.1038/s41401-018-0036-4 PMC6289389

[35] Harvey KA , Welch Z , Sliva D , Siddiqui RA . Role of Rho kinase in sphingosine 1‐phosphate‐mediated endothelial and smooth muscle cell migration and differentiation. Mol Cell Biochem. 2010;342:7‐19. 20401628 10.1007/s11010-010-0461-2

[36] Park KS , Kim M‐K , Lee HY , et al. S1P stimulates chemotactic migration and invasion in OVCAR3 ovarian cancer cells. Biochem Biophys Res Commun. 2007;356:239‐244. 17349972 10.1016/j.bbrc.2007.02.112

[37] Brizuela L , Rábano M , Gangoiti P , et al. Sphingosine‐1‐phosphate stimulates aldosterone secretion through a mechanism involving the PI3K/PKB and MEK/ERK 1/2 pathways. J Lipid Res. 2007;48:2264‐2274. 17609523 10.1194/jlr.M700291-JLR200

[38] Panupinthu N , Rogers JT , Zhao L , et al. P2X7 receptors on osteoblasts couple to production of lysophosphatidic acid: a signaling axis promoting osteogenesis. J Cell Biol. 2008;181:859‐871. 18519738 10.1083/jcb.200708037 PMC2396816

[39] Park MY , Kim HS , Lee M , et al. FAM19A5, a brain‐specific chemokine, inhibits RANKL‐induced osteoclast formation through formyl peptide receptor 2. Sci Rep. 2017;7:15575. 29138422 10.1038/s41598-017-15586-0 PMC5686125

[40] Kim JH , Kim N . Signaling pathways in osteoclast differentiation. Chonnam Med J. 2016;52:12‐17. 26865996 10.4068/cmj.2016.52.1.12 PMC4742606

[41] Kovacs E , Harmat V , Tóth J , et al. Structure and mechanism of calmodulin binding to a signaling sphingolipid reveal new aspects of lipid‐protein interactions. FASEB J. 2010;24:3829‐3839. 20522785 10.1096/fj.10-155614 PMC2996911

[42] Aliprantis AO , Ueki Y , Sulyanto R , et al. NFATc1 in mice represses osteoprotegerin during osteoclastogenesis and dissociates systemic osteopenia from inflammation in cherubism. J Clin Invest. 2008;118:3775‐3789. 18846253 10.1172/JCI35711 PMC2564610

[43] Komori T . Animal models for osteoporosis. Eur J Pharmacol. 2015;759:287‐294. 25814262 10.1016/j.ejphar.2015.03.028

[44] Cao JJ , Gregoire BR , Sun L , Song S . Alpha‐1 antitrypsin reduces ovariectomy‐induced bone loss in mice. Ann NY Acad Sci. 2011;1240:E31‐E35. 22360827 10.1111/j.1749-6632.2011.06370.x

[45] Liu R , Farach‐Carson MC , Karin NJ . Effects of sphingosine derivatives on MC3T3‐E1 pre‐osteoblasts: psychosine elicits release of calcium from intracellualr stores. Biochem Biophys Res Commun. 1995;214:676‐684. 7677781 10.1006/bbrc.1995.2339

[46] Riggs BL . The mechanisms of estrogen regulation of bone resorption. J Clin Invest. 2000;106:1203‐1204. 11086020 10.1172/JCI11468 PMC381441

[47] Liu L , Zhou L , Yang X , et al. 17β‐estradiol attenuates ovariectomy‐induced bone deterioration through the suppression of the ephA2/ephrinA2 signaling pathway. Mol Med Rep. 2017;17:1609‐1616. 29138859 10.3892/mmr.2017.8042 PMC5780101

[48] Vozella V , Basit A , Piras F , et al. Elevated plasma ceramide levels in post‐menopausal women: a cross‐sectional study. Aging. 2019;11:73‐88. 30620722 10.18632/aging.101719 PMC6339790

[49] Flammier S , Peyruchaud O , Bourguillault F , et al. Osteoclast‐derived autotaxin, a distinguishing factor for inflammatory bone loss. Arthritis Rheumatol. 2019;71:1801‐1811. 31162832 10.1002/art.41005 PMC6817375

